# Prognostic Value of PCO_2_ Gap in Adult Septic Shock Patients: A Systematic Review and Meta-Analysis

**DOI:** 10.5152/TJAR.2021.21139

**Published:** 2022-10-01

**Authors:** Prihatma Kriswidyatomo, Yudhistira Pradnyan Kloping, Merlin Guntur Jaya, Ricardo Adrian Nugraha, Corinne Prawira Putri, Dana Hendrawan Putra, Nabila Ananda Kloping, Taufan Adityawardhana, Niwanda Yogiswara, Nancy Margarita Rehatta

**Affiliations:** 1 Post Graduate Doctoral Program, Faculty of Medicine Universitas Airlangga, Surabaya, Indonesia; 2 Universitas Airlangga Faculty of Medicine, Surabaya, Indonesia; 3 Department of Cardiology, Universitas Airlangga Faculty of Medicine, Surabaya, Indonesia; 4 Department of Medical Education Research and Staff Development, Universitas Airlangga Faculty of Medicine, Surabaya, Indonesia; 5 Department of Anaesthesiology and Reanimation, Universitas Airlangga Faculty of Medicine, Surabaya, Indonesia

**Keywords:** PCO_2_ gap, sepsis, septic shock, venous-arterial PCO_2_ difference

## Abstract

Sepsis cases caused substantial mortality and a significant burden on healthcare costs and resources. To tackle this problem, there has been discussion surrounding O_2_ parameters as it has a distinct outcome in septic patients. This review aimed to evaluate the prognostic value of the central venous-arterial carbon dioxide difference (PCO_2_) gap in patients with septic shock. A comprehensive systematic search was performed through electronic databases including Pubmed, Scopus, and Embase for studies focusing on the use of PCO_2_ gap as a mortality predictor in septic shock patients. Other secondary outcomes such as mean arterial pressure, lactate clearance, the acute physiology and chronic health evaluation II score, and intensive care unit length of stay were also measured. The Newcastle–Ottawa Scale tool was used to assess the risk of bias. A total of 8 studies were analysed. The mortality rate (odds ratio = 0.50, 95% CI = 0.28-0.87, *P* < .01) and lactate levels (mean difference [MD] = −0.98; 95% CI = −1.62 to −0.35; *P* = .001) of the low PCO_2_ gap group were significantly lower than the high gap group. The low gap group had a significantly higher mean arterial pressure compared to the high gap group (MD = 4.54; 95% CI = 2.14 to 6.95; *P* = .001). There were no pronounced outcomes in acute physiology and chronic health evaluation score and intensive care unit length of stay. PCO_2_ gap can potentially be used as a marker for mortality rate in septic shock patients. It is also significantly associated with other predictors, such as mean arterial pressure and lactate clearance.

**Main Points**

- PCO_2 _gap higher than 6 mm Hg had a higher mortality rate.
- PCO_2 _is a potential marker for establishing a prognosis for septic shock patients.
- As a prognostic marker, the timing of PCO_2_ measurement is crucial.

## Introduction

Sepsis remains to be a major healthcare issue worldwide as it causes substantial mortality and a significant burden on healthcare costs and resources. Numerous studies have highlighted the increasing prevalence and incidence of sepsis globally.^1-4^ There are several guidelines and consensus made to establish the standards of care for sepsis patients. However, there was still a high mortality rate of septic patients in Turkey even after being admitted to an intensive care unit (ICU).^5^ Critical patients with multiple organ failures, including circulatory and metabolic dysfunctions, have a significantly higher mortality rate. Optimizing haemodynamics, improving metabolic status, and maintaining adequate tissue oxygenation are crucial in critically ill patients.^6^ Several parameters have been utilized to assess the adequacy of tissue oxygen (O_2_) requirements. Clinical examinations, lactate, and central or mixed venous O_2_ saturation have their respective limitations in assessing adequate tissue oxygenation.^7^ Oxygen-derived parameters have been questioned due to distinct outcomes in sepsis patients from several recent studies, and thus a different parameter of evaluation is needed. A variable that has been newly proposed as a parameter for cardiac output relative to metabolic demand is the central venous-arterial carbon dioxide difference (PCO_2_ gap).^8^ PCO_2_ gap indicates the difference between partial pressure of carbon dioxide (CO_2_) in central venous blood (PcvCO_2_) and arterial blood (PaCO_2_). Carbon dioxide is a more sensitive marker of hypoperfusion compared to O_2_ as it reliably diffuses out to the venous blood from ischemic tissues.^9^ PCO_2_ gap is inversely related to cardiac output as mentioned by several evidence.^10^ In an attempt to overcome limitations from previous variables, prognostic value from PCO_2_ gap is proposed to bring a more reliable prediction of adequate tissue O_2_ supply and requirements in sepsis. However, there have been reports showing inconsistent findings regarding its role as a reliable marker.^11,12^ Therefore, in this meta-analysis, we aimed to evaluate the prognostic value of PCO_2_ gap in patients with septic shock.

## Methods

This systematic review was conducted according to the Cochrane Handbook for Systematic Review of Interventions^13^ and was based on Preferred Reporting Items for Systematic Reviews and Meta-Analysis.^14^ This systematic review and meta-analysis have been registered in the Prospective Register of Systematic Reviews (PROSPERO) public database (CRD42020210399).

All independent authors conducted the computerized data search through databases including Pubmed, Scopus, and Embase. Keywords based on medical subject heading (MeSH) terms were used. The combination of keywords consists of “hypercapnia” OR “hypercarbia” OR “carbon” AND “dioxide” OR “pCO_2_” OR “pCO_2_ gap” AND “septic” AND “shock”.

We determined the eligibility criteria using the population, intervention, comparison, and outcomes model. The samples were adult patients with septic shock whose PCO_2_ gap was evaluated using blood gas analysis. PCO_2_ gap was assessed thoroughly by various outcomes including 1 main prognostic outcome and several secondary outcomes. The main prognostic outcome of this study was mortality rate, whereas the secondary outcomes mean arterial pressure (MAP), lactate clearance, the acute physiology and chronic health evaluation (APACHE II) score, and ICU length of stay (LOS). The inclusion criteria were as follows: (1) adult human samples diagnosed with septic shock; (2) observational prospective cohort, retrospective cohort, and cross-sectional studies; and (3) studies published in English. The criteria used to exclude studies were as follows: (1) the study was duplicated or redundant publication; (2) study was based on in vitro models, animal models, or paediatric samples; (3) other aetiologies of shock; and (4) review, editorials, correspondence, and case report/case series studies.

### Statistical Analysis

Data assessment and quality assessments of each included studies were performed by independent investigators to determine its eligibility. Risk of bias quality assessment was performed using the Newcastle–Ottawa Scale for observational studies.^15^ Statistical analysis and data synthesis were performed using STATA 16.0 software (StataCorp LLC). The data were extracted based on author, year of publication, study design, sample size, timing of PCO_2_, MAP, lactate difference, overall mortality, APACHE score, and ICU LOS. Fixed-effects model was used in studies with low statistical heterogeneity (*I*^2^ < 50%). Otherwise, random-effects model would be used (*I*^2^ > 50%). We assessed potential bias in the published literature using a funnel plot.

## Results

**Figure 1.**
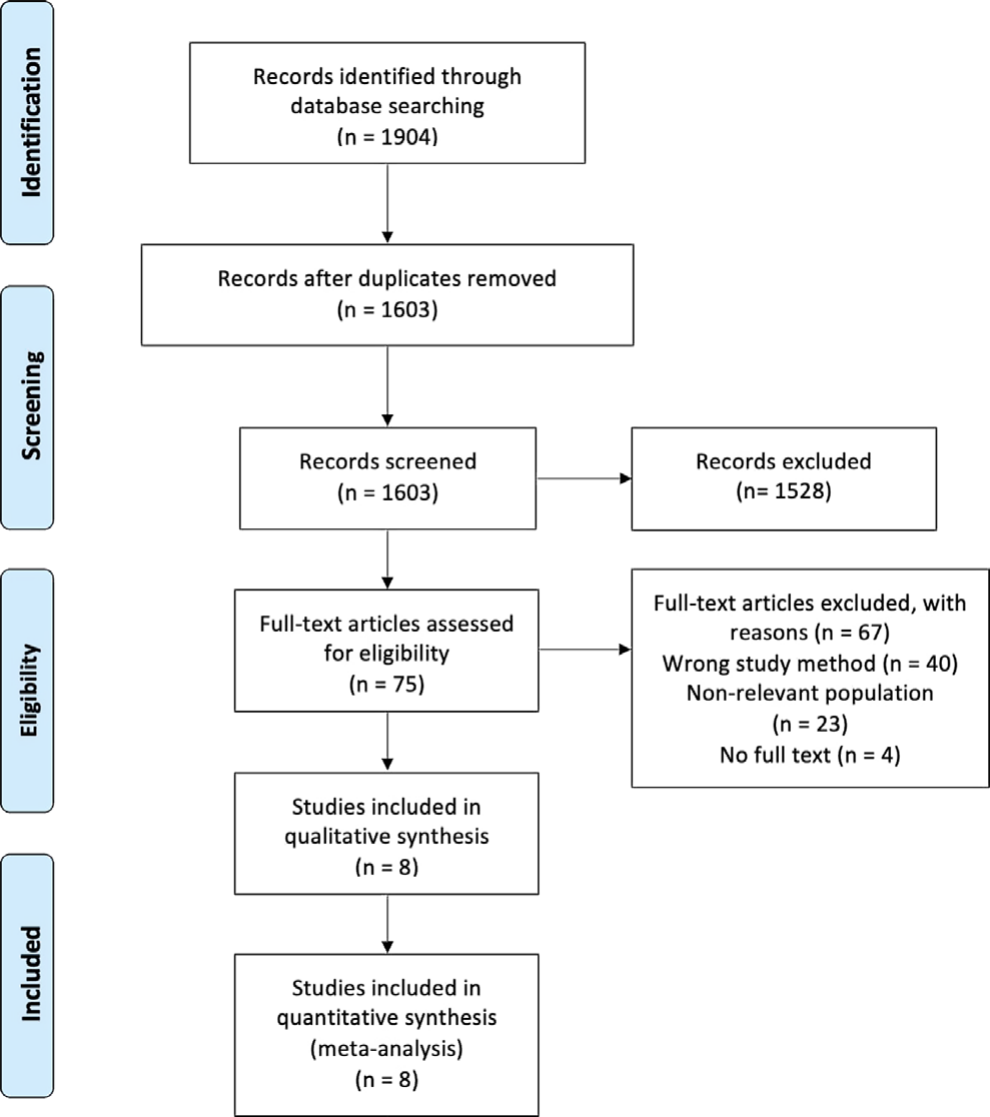
Preferred Reporting Items for Systematic Reviews and Meta-Analysis flowchart displaying how the studies were included.

We found 1904 articles through the systematic search process shown in Figure 1. After removing duplicates and screening the articles, 8 studies were considered eligible for meta-analysis.^6,11,12,16-20^ There was a total of 503 subjects pooled with the majority of male patients. The mean age of all participants in the low gap group was 51.3-69 years old, while in the high gap group it was 53-73.62 years old. Table 1 presents the summary of the included studies. The risk of bias assessment of the studies is shown in Table 2.

**Figure 2.**
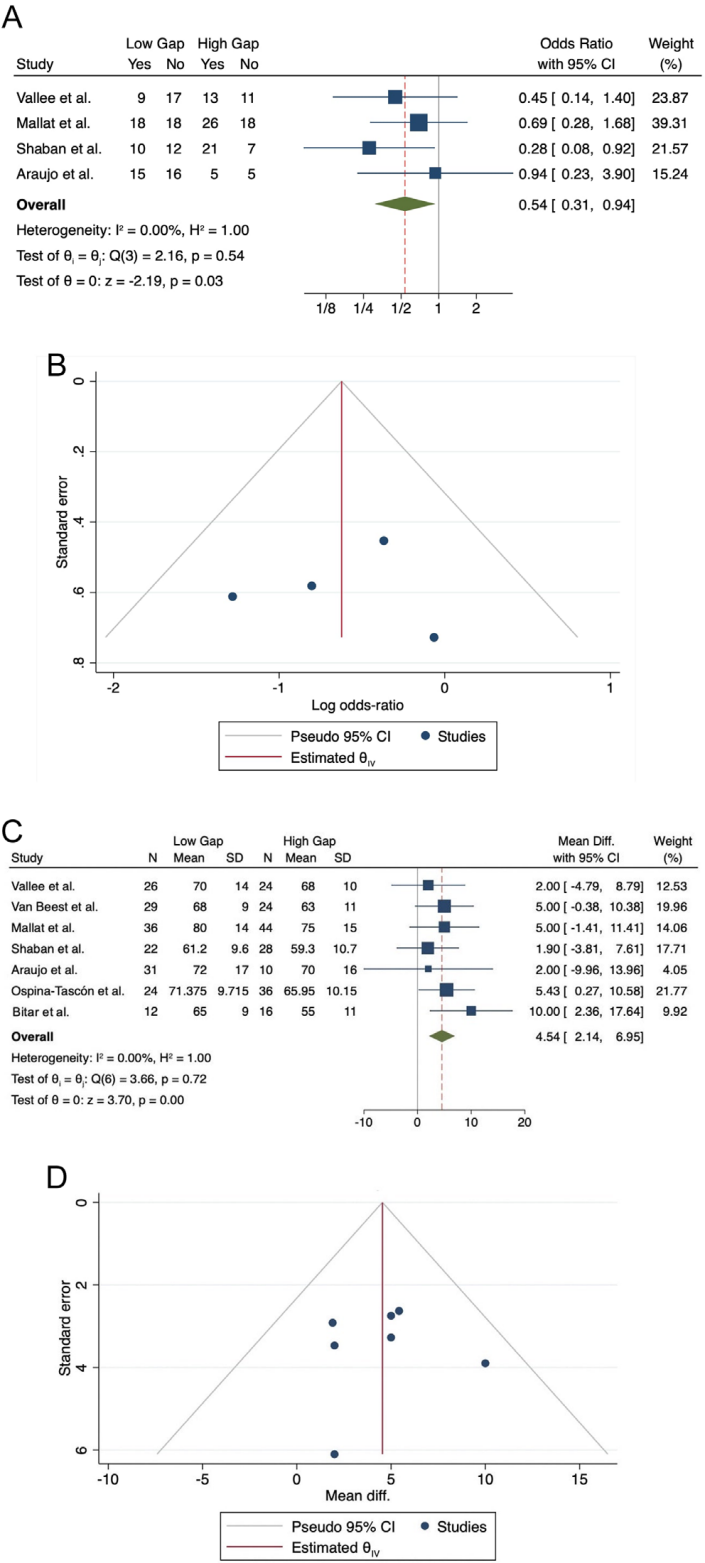
(A) Forest plot of pooled data on 28-day mortality, (B) funnel plot on 28-day mortality, (C) forest plot of pooled data on mean arterial pressure (MAP), (D) funnel plot on MAP in the low PCO_2_ gap group versus the high PCO_2_ gap group measured at admission.

**Figure 3.**
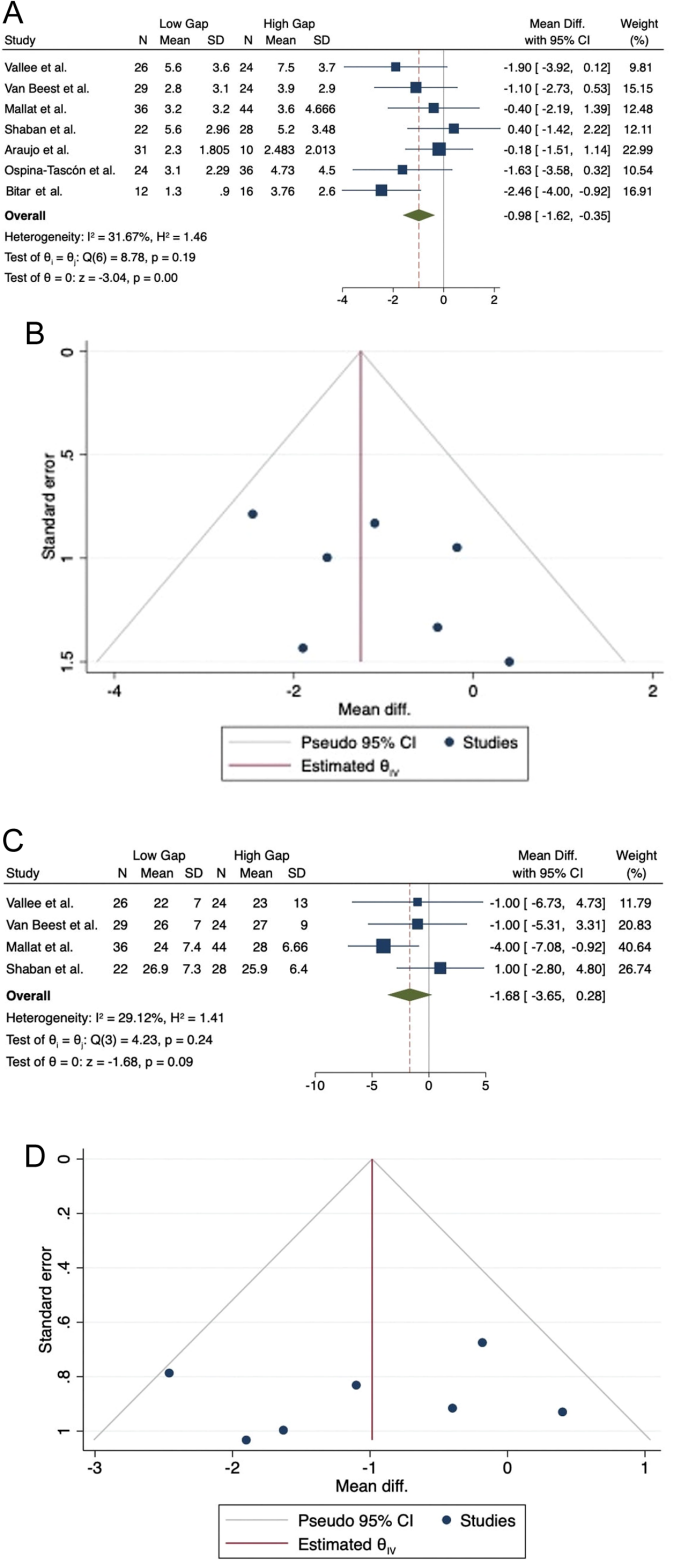
(A) forest plot of pooled data on lactate levels, (B) funnel plot on lactate levels, (C) forest plot of pooled data on APACHE II, (D) funnel plot on APACHE II in the low PCO_2_ gap group versus the high PCO_2_ gap group measured at admission. APACHE II, acute physiology and chronic health evaluation II.

All studies’ definition of PCO_2 _is the same with 0.8 kPa equal to 6.00049 mm Hg. The timing of PCO_2 _measurement was divided into admission and 6 hours post-resuscitation. According to the analysis, all forest plots used fixed-effect model, except for one forest plot (ICU LOS) because of its low heterogeneity. According to the *P*-value, there were no publication bias found from each funnel plot (Figure 2B, *P* = .9026, Figure 2D, *P* = .9822, Figure 3B, *P* = .6730, Figure 3D, *P* = .4758).

## Early Resuscitation

### Twenty-Eight-Day Mortality Rate

Four studies with a total of 221 participants evaluating the 28-day mortality rate were enrolled in this meta-analysis. The crude rate of mortality was higher in the high gap group (46%-75%) compared to the low gap group (35%-50%). Pooled data revealed that the mortality rate of the low gap group was significantly lower than the high gap group (odds ratio (OR) = 0.54, 95% CI = 0.31-0.94, *P* < .03) as shown in Figure 2.

### Mean Arterial Pressure

The differences in MAP were pooled from 7 studies with a total of 362 participants. Pooled data in Figure 2 revealed that the low gap group had significantly higher MAP compared to the high gap group (mean difference [MD] = 4.54; 95% CI = 2.14-6.95; *P* = .001).

### Lactate Levels

The difference in the lactate concentration of 362 participants from 7 studies between the low and high gap groups was summarized as a forest plot presented in Figure 3. The analysis demonstrated that there was a significantly lower lactate in the low gap group compared to the high gap group (MD = -0.98; 95% CI = -1.62 to -0.35; *P* = .001).

**Acute Physiology and Chronic Health Evaluation II** Pooled data of the APACHE II Score of 233 participants from 4 studies showed no difference between the low gap group and the high gap group (MD = -1.68; 95%s CI = -3.65 to 0.28; *P* = .09).

### Intensive Care Unit Length of Stay

**Figure 4.**
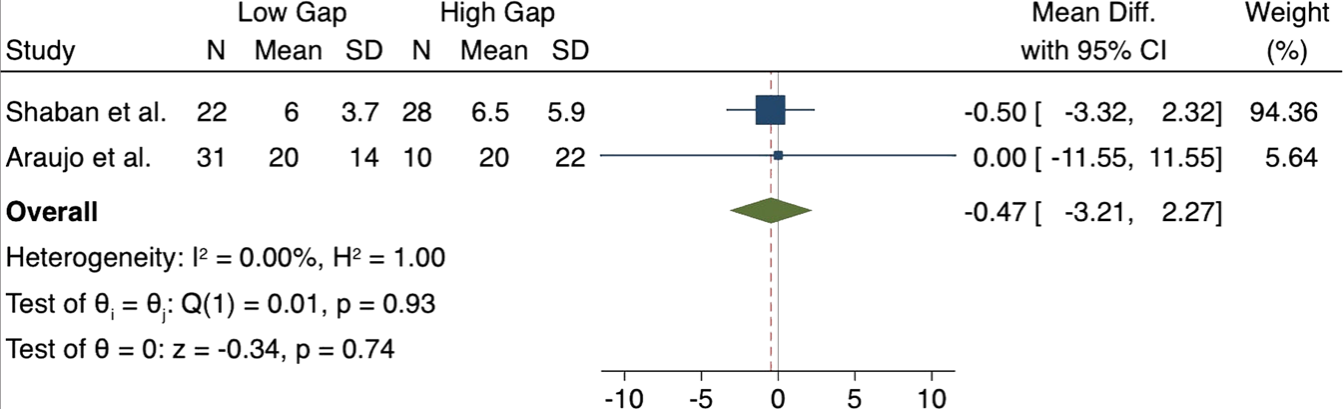
Pooled data of mean differences in the low PCO_2_ gap group versus the high PCO_2_ gap group measured at admission for ICU LOS.ICU LOS, intensive care unit length of stay.

Figure 4 presents the pooled estimate mean differences of ICU LOS from 2 studies. Pooled data showed no significant differences between the low gap group and high group in terms of ICU LOS (MD = -0.44; 95% CI = -3.04 to 2.16; *P* = .74).

## Six Hours Post Resuscitation

### Mean Arterial Pressure

**Figure 5.**
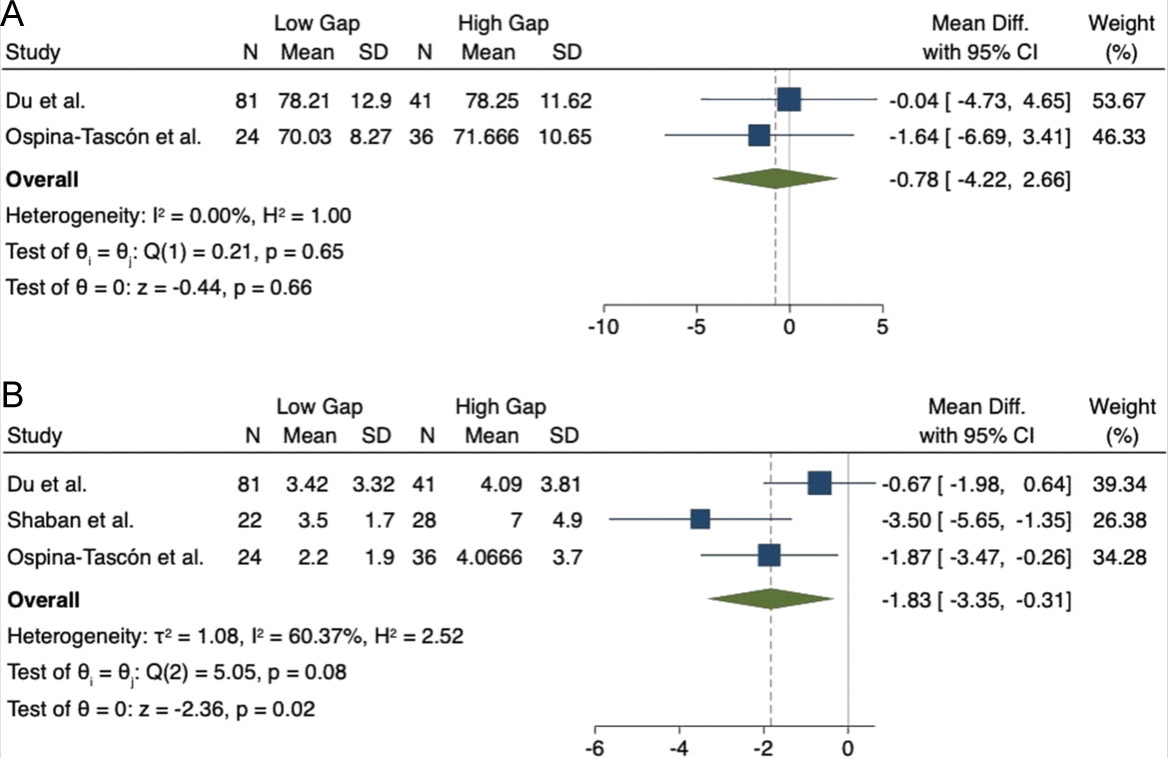
Pooled data in the low PCO_2_ gap group versus the high PCO_2_ gap group measured at 6 hours. (A) Mean arterial pressure, (B) lactate levels.

The mean differences in MAP after 6 hours of admission were pooled from 2 studies with a total of 182 participants. Pooled data in Figure 5 revealed that the MAP in the low gap group had no significant value compared to the high gap group (MD = -0.78; 95% CI = -4.22 to 2.66; *P* = .66).

### Lactate Levels

The mean difference in the lactate concentration after 6 hours of admission in 232 participants from 3 studies between the low and high gap groups was summarized as a forest plot displayed in Figure 5. The analysis demonstrated that there was significantly lower lactate in the low gap group compared to the high gap group (MD = -1.83; 95% CI = -3.35 to -0.31; *P* = .02).

## Discussion

This study reviewed 8 prospective cohort studies focusing on the role of PCO_2_ gap as a marker in septic shock patients. Previous studies discovered that O_2_ parameters in sepsis patients were normalized after ICU admission, indicating that early global resuscitation might be achieved.^21,22^ Several studies pushed early goal-directed therapy as strategy guidance; however, these studies initiated the strategy after volume repletion with close to normal O_2_ saturation at randomization, indicating that the restoration of blood flow had already been achieved.^23^ Therefore, mixed or venous O_2_ saturation normal values may not represent tissue perfusion abnormalities.^16,24^ Hypoperfusion of tissues during circulatory failure due to various aetiologies of shock is associated with increased PCO_2_. Mixed venous hypercapnia indicates inadequate clearance of CO_2_ due to cellular oxidative and buffering systems’ processes. This accumulation translates into an increase of mixed venous-to-arterial carbon dioxide gradient or PCO_2_ gap.^17^ The discovery that PCO_2_ gap is able to track blood flow tissues leads to studies evaluating its role in sepsis and septic shock patients.^19^

### Twenty-Eight-Day Mortality Rate

Mortality is commonly associated with sepsis and septic shock. Due to its high mortality rate, early recognition of clinical deterioration is necessary.^25^ The shift from systemic inflammatory syndrome to severe sepsis and eventually septic shock involves several pathogenic alterations including circulatory abnormalities resulting in global tissue hypoxia.^26^ The prognostic value of PCO_2_ gap has been suggested by several studies based on its ability to predict adverse clinical outcomes among septic shock patients with normal O_2_-derived parameters during the early phases of resuscitation.^6^ The 4 included studies in this review mostly showed the promise of PCO_2_ gap to predict the mortality rate of septic shock patients. One included study claimed that the mortality rate between the groups was not significantly different.^20^ However, the other studies highlighted the significantly lower mortality rate among the low PCO_2_ gap group compared to the high PCO_2_ gap group. A high PCO_2_ gap could therefore indicate poor prognosis due to severe microcirculatory dysfunction.^6^ In sepsis patients, microcirculatory level distributive changes may be independent of cardiac index as the accumulation of CO_2_ may occur in sepsis due to persistent tissue hypoxia despite normal central venous O_2_ saturation levels.^10,26,27^

### Mean Arterial Pressure

Increases in venous-to-arterial CO_2_ gap has been used as a marker of peripheral hypoperfusion in both septic and cardiogenic shock.^28^ Restoration of the MAP is one of the most important initial goals for resuscitation and is a common target to restore end-organ perfusion. However, haemodynamic restoration does not always guarantee end-organ perfusion. In order for perfusion to be adequate, the oxygen delivery must meet the demands of cellular O_2_ consumption where PCO_2_ gap can be used as a surrogate for microcirculatory blood flow with several limitations. PCO_2_ gap may provide valuable clinical information to determine the adequacy of both perfusion and cardiac output.^9^ In the treatment of patients with septic shock, increasing MAP above 65 mm Hg with a higher dose of norepinephrine administration resulted in improved microcirculatory function, including the PCO_2_ gap.^29^ Sufficient MAP maintenance to avoid tissue hypoperfusion is key in the management of distributive shock, and after the MAP is sustainable.^30^ On the other hand, when PCO_2_ and MAP measured 6 hours after resuscitation, they are not associated. This could mean that after 6 hours, MAP portrayed the restored peripheral perfusion. PCO_2_ gap can potentially be used as a reliable prediction for measuring/maintaining the adequacy of tissue O_2_ supply and requirements in shock patients when measured after early resuscitation.

### Lactate Levels

Lactate level changes represent the sum of ongoing production and removal from the blood by means of excretion by urine or sweat and its metabolism.^31^ Increased levels of lactate are associated with circulatory dysfunction. In septic patients, lactate levels increase or ongoing hyperlactataemia may indicate decreased clearance rather than an increase in production.^32^ There are many findings that indicate lactate as a marker of illness severity. It is considered a powerful mortality predictor.^33^ Early lactate clearance in septic patients is believed to be associated with improved survival.^34^ However, the complexity of lactate as a molecule, marker, energy source, and modulator of cellular bioenergetics makes it impossible to determine what it should be a target of. Lowering lactate levels has no purpose and logic in haemodynamic or tissue protection terms. On the other hand, assisting the natural process of lactate generation and utilization during sepsis makes more sense.^35^ The analysis of the 7 included studies demonstrated in this review showed significantly lower lactate levels in the low PCO_2_ gap group indicating a higher possibility of survival in the low gap group compared to the high gap group when measured just after resuscitation (Figure 3A). Moreover, we found that PCO_2 _gap is still significantly associated with lower lactate levels when measured 6 hours after resuscitation. This means that a low PCO_2 _gap is a potential marker for survival that could be used alongside lactate. However, a study by Van Beest et al^11^ claimed that a high PCO_2_ gap difference at baseline was inversely correlated with lactate clearance and reduction in SOFA score after 24 hours. A similar result was discovered by Vallee et al^20^ who claimed that the clearance of lactate was significantly larger among the low gap group than in the high gap group. On the contrary, one of the included studies by Araujo et al^19^ discovered that there were no differences in lactate levels between the PCO_2_ gap groups at the time of patient admission. They concluded that PCO_2_ gap is not a marker of tissue hypoxia, indicated by parameters such as lactate, but by the adequacy of blood flow to remove tissue CO_2_. Hyperlactataemia and its association with PCO_2_ gap in sepsis are complex since lactate accumulation may occur during accelerated aerobic veneration or slow clearance.

## Acute Physiology and Chronic Health Evaluation II

In this review, there is no difference between the low gap group and the high gap group in the included 7 studies. This could be because APACHE II was sensitive to interventions, in which most patients in the included studies underwent various therapies. Du et al^16^ and Shaban et al^18^ discovered that higher APACHE II scores were reciprocal with the mortality rate indicating a positive association between the 2. However, it is not associated with PCO_2_ gap as a predictor.

### Intensive Care Unit Length of Stay

Based on the pooled data from studies included in this review, ICU-LOS is not significantly associated with PCO_2_ gap levels. Clinicians typically utilize ICU-LOS predictors for planning ICU capacity, identifying unexpectedly long ICU LOS, and ICU benchmarking.^36^ The association between severity of illness and ICU-LOS differs for ICU survivors and ICU non-survivors, and thus it may lead to wildly inaccurate or biased predictions of clinical outcome or development if not used properly. It is even concluded that the currently available models for ICU-LOS are not suitable for predicting individual patient data.^36,37^ Even though mortality rates increase with increasing LOS, there does not appear to be a clear cut-off point when a patient’s prognosis changes.^38^ Intensive care unit length of stay is difficult to be accurately correlated with an individual’s clinical evaluation, let alone markers, such as PCO_2_ gap.

There are a few limitations in this meta-analysis. The compiled studies presented various different results. There are concerns about the generality of the evidence. Moreover, in this review, only studies in English were included, which might limit additional findings in other languages. At the moment, studies on PCO_2_ are limited to observational studies and reviews. Clinical trials with a uniform method are needed to prevent heterogeneity between studies.

Apart from the PCO_2_ gap, taking an oxygen-derived variable like arterial-to-central venous O_2 _content difference (Ca-vO_2_) into account may also be useful in septic shock patients. Measuring both by assessing the ratio between PCO_2_ gap and Ca-vO_2_ may potentially be reliable as another predictor of survival and clinical outcomes of septic shock as it combines both CO_2_ and O_2_ variables.^18^ Ultimately, the ideal marker for critically ill patients in the ICU should be simple to measure, interpret, adaptable for treatment, and not invasive.^39^ The prospects of PCO_2_ gap as a reliable predictor found in this review may be used to provide supplementary information in generating strategies for managing septic shock in the future.

## Conclusion

PCO_2_ gap can potentially be used as a marker for mortality rate in septic shock patients. In relation to other parameters, it is significantly associated after early resuscitation with MAP and lactate clearance, but only if lactate was measured 6 hours post-resuscitation.

## Figures and Tables

**Table 1.** Characteristics of the Included Studies

**Author**	**Year**	**Study Design**	**Region**	**PCO2 (n)** **Low/High Gap**	**Patients Profile** **Low/High Gap** **[Age (Years), Male (n)]**	**Study Definition of PCO2**		**Timing of PCO2 Measurement**
**Low Gap**	**High Gap**
Bitar et al^6^	2020	Prospective cohort	Kuwait	12/16	69 ± 17/73.62 ± 13, 7/10	<0.8 kPa*	>0.8 kPa*	T0 and T6 after CCU admission.
Van Beest et al^11^	2013	Retrospective cohort	Europe	29/24	67 ± 13/66 ± 11,17/11	<0.8 kPa*	>0.8 kPa*	Post resuscitation (6 hours)
Ospina-Tascón et al^12^	2013	Prospective cohort	America	24/36	62/63, 22/17	<6 mm Hg	≥6 mm Hg	First 6 hours of resuscitation
Du et al^16^	2013	Retrospective cohort	Asia	81/41	59 ± 18/62 ± 1,51/27	<6 mm Hg	≥6 mm Hg	Post resuscitation (6 hours)
Mallat et al^17^	2014	Prospective cohort	Europe	36/44	N/A	≤0.8 kPa*	>0.8 kPa*	First 6 hours of resuscitation
Shaban et al^18^	2017	Prospective cohort	Asia	22/28	58 ± 19/53 ± 20,12/15	<6 mm Hg	≥6 mm Hg	T0 (ICU admission) is defined as post-resuscitation
Araujo et al^19^	2019	Prospective cohort	America	31/10	56 ± 13/60 ± 15,N/A	≤6 mm Hg	>6 mm Hg	After 2 hours of EGDT completion
Vallee et al^20^	2008	Prospective cohort	Europe	26/24	51 ± 13/55 ± 20,16/14	<6 mm Hg	≥6 mm Hg	Post early resuscitation in the ICU

*0.8 kPa = 6.00049 mm Hg.

EGDT, early goal-directed therapy; CCU, critical care unit; N/A, not available.

**Table 2.** Newcastle–Ottawa Scale Used for Assessing the Quality of the Included Studies

**Author**	**Selection**				**Comparability**		**Outcome**			**NOS Score**	**Interpretation of Quality**
	**Representativeness of the Exposed Cohort**	**Selection of the Non-Exposed Cohort**	**Ascertainment of Exposure**	**Demonstration That Outcome of Interest Was Not Present at Start of Study**	**Comparability of Cohorts on the Basis of the Design or Analysis**		**Assessment of Outcome**	**Was Follow-Up Long Enough for Outcomes to Occur?**	**Adequacy of Follow-Up of Cohorts**		
**Age and Sex Adjustment**	**Additional Factors Adjustment**
Bitar et al^6^	*		*	*	*	*	*		*	7	Good
Van Beest et al^11^	*		*		*	*	*			5	Poor
Ospina-Tascón et al^12^	*		*	*	*	*	*	*	*	8	Good
Du et al^16^	*		*		*	*	*	*	*	7	Good
Mallat et al^17^	*		*	*	*	*	*	*	*	8	Good
Shaban et al^18^	*		*	*	*	*	*	*	*	8	Good
Araujo et al^19^	*		*	*	*	*	*	*	*	8	Good
Vallee et al ^20^	*		*	*	*	*	*	*	*	8	Good

NOS, Newcastle–Ottawa Scale.

* = item was found in text.
